# Evaluation of Stress–Strain Behavior of Self-Compacting Rubber Lightweight Aggregate Concrete under Uniaxial Compression Loading

**DOI:** 10.3390/ma12244064

**Published:** 2019-12-05

**Authors:** Jing Lv, Tianhua Zhou, Qiang Du, Kunlun Li, Kai Sun

**Affiliations:** 1 School of Civil Engineering, Chang’an University, Xi’an 710061, China; zhouth@chd.edu.cn (T.Z.); 2017128074@chd.edu.cn (K.L.); 2017128024@chd.edu.cn (K.S.); 2 School of Economics and Management, Chang’an University, Xi’an 710064, China; q.du@chd.edu.cn

**Keywords:** self-compacting rubber lightweight aggregate concrete, stress–strain relationship, uniaxial compressive experiments, predictive model

## Abstract

The recycling of waste tires in lightweight aggregate concrete (LC) would achieve huge environmental and societal benefits, but the effects of rubber particles on the partial properties of LC are not clear (e.g., the stress–strain relationship). In this paper, uniaxial compressive experiments were conducted to evaluate the stress–strain relationship of self-compacting rubber lightweight aggregate concrete (SCRLC). Rubber particles were used to replace sand by volume, and substitution percentages of 0%, 10%, 20%, 30%, 40%, and 50% were set as influence factors. Experimental results indicate that with increased rubber particles substitution percentage, the cubic compressive strength and axial compressive strength of SCRLC decreased, while the failure modes of SCRLC prism specimens gradually changed from brittle to ductile failure. As the rubber particles substitution percentage increased from 0% to 50%, the peak strain of SCRLC increased whereas peak stress, elastic modulus, and peak secant modulus of SCRLC deceased, the descending stage of stress–strain curves became softer. The rubber particles substitution percentage of 30% was the critical point at which an obvious change in the properties of SCRLC occurred. Based on the data collected from experimental studies, a predictive model for SCRLC was established and a further prediction of the SCRLC stress–strain relationship was given.

## 1. Introduction

The large number of waste tires has caused many environmental issues in recent years [1]. According to the statistics [2,3,4], the global tire demand reached 2.9 billion tires in 2017 and more than 1 billion tires have been discarded due to a lack of appropriate treatment technology. With the rapid development of automotive industry, this issue will become increasingly prominent. Previous studies indicate that crushing waste tires into particles and applying them in concrete is an effective way to recycle them [5]. Numerous benefits can be achieved, such as reducing the environmental effect of waste tires, improving the partial properties of concrete, saving natural sand resources and more [6,7,8]. The depletion of natural resources and aggravation of environmental pollution make the reclamation of waste tires an urgent issue in the future, especially for developing countries. For example, in order to protect the Qinling mountains in Shaanxi Province, China, the exploitation of natural sand and gravel in the mountains are prohibited as of 2018. This has resulted in a serious shortage of natural sand and gravel in Xi’an city. Therefore, crushing waste tires into particles and using them as fine aggregate is a beneficial way to recycle waste tires, and will achieve huge environmental and societal benefits in future.

So far, many investigations have been carried out on the properties of normal concrete (NC) using rubber particles as fine aggregate [9,10,11,12]. The results indicate that with increased fraction of rubber particles in NC, the flowability, self-weight, compressive strength, flexural strength, splitting tensile strength, and elastic modulus drop, while toughness enhances [13,14,15,16,17]. Concrete containing rubber particles has also been successfully applied in structures [18,19,20,21,22,23,24]. According to the research experience of normal rubberized concrete, rubber particles can be also utilized in lightweight aggregate concrete (LC), which has been widely used in the past few years [25,26,27,28,29]. The literature indicates that rubber particles have similar effects on the properties of LC and NC [28,29]. One problem is that the incorporation of rubber particles in LC leads to a much more serious separation of coarse aggregate during the vibration process. In order to reduce the separation of aggregate in rubber lightweight aggregate concrete (RLC), self-compacting rubber lightweight aggregate concrete (SCRLC) has been proposed [29]. Although many studies have focused on the self-compacting performance of NC and recycled concrete [30,31,32], it is still a challenge to achieve self-compacting in RLC. Lv et al. [29] investigated the fresh and mechanical properties of SCRLC with rubber particles substitution percentages of 0%, 10%, 20%, 30%, 40%, and 50%, and reported that RLC could successfully achieve self-compaction with reasonable mix proportion design. Although few studies have focused on the mechanical properties of hardened SCRLC, numerous studies on the mechanical properties of RLC provide guidance for SCRLC.

Nevertheless, in order to popularize and utilize a new structural material in structures, it is insufficient to understand only its fresh and mechanical behaviors; it is also necessary to understand its nonlinear behavior in order to guide the structural design. The stress–strain relationship of SCRLC is a nonlinear parameter that plays a crucial role in the design of reliable SCRLC structures [33,34]. However, few existing studies have been conducted on the stress–strain behavior of SCRLC. This will affect the accuracy of the force analysis of SCRLC structures and limit the popularization and application of SCRLC in structural members.

To gain a better understanding of the stress–strain relationship of SCRLC with various rubber particle contents, experimental studies and analysis on the stress–strain curves of SCRLC were carried out in this research. Using uniaxial compressive tests, stress–strain curves of SCRLC with rubber particles substitution percentages of 0%, 10%, 20%, 30%, 40%, and 50% were collected. After analysis, the relationships between parameters detected from the SCRLC stress–strain curves were established. Based on the comparison of existing widely used stress–strain relationship models of NC with the experimental SCRLC stress–strain curves, a more suitable stress–strain relationship model for SCRLC is proposed. In combination with the established relationships between characteristic parameters, the stress–strain relationships of SCRLC at different strength grades are predicted.

## 2. Experimental Program

### 2.1. Material Properties 

**Figure 1:**
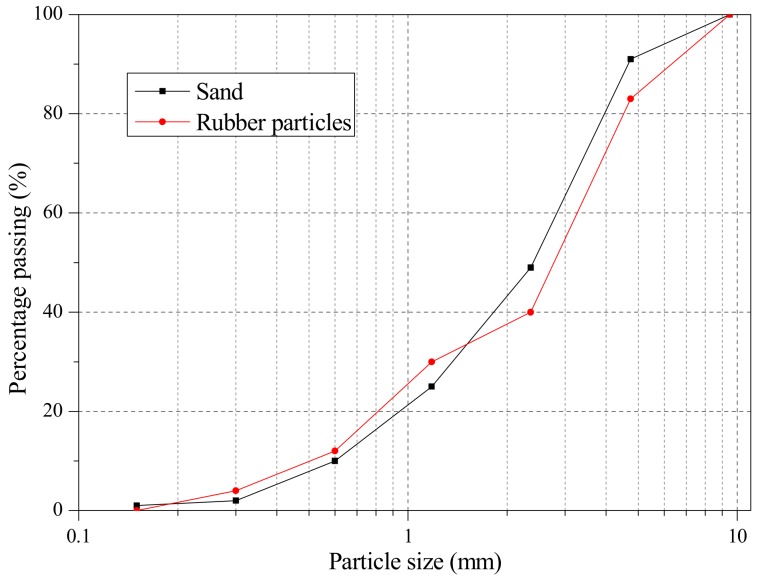
Particle size distribution of sand and rubber particles.

The SCRLC investigated in this research was composed of ordinary Portland cement, class I fly ash, shale ceramsite, river sand, rubber particles, thickening agent, polycarboxylate superplasticizer, and tap water. The chemical compositions of ordinary Portland cement and fly ash are shown in Table 1. The shale ceramsite was crushed shale ceramsite with a crushing strength of 8.82 MPa, bulk density of 842 kg/m^3^, and particle size from 4.75 to 19 mm. The rubber particles were manufactured by mechanically shredding waste tires, and were utilized to replace sand as a fine aggregate in SCRLC by volume. The rubber particles were prepared with a similar size gradation of river sand (as shown in Figure 1). The physical properties of sand were: fineness modulus 2.8, density 2652 kg/m^3^, and bulk density 1425 kg/m^3^. The physical properties of the rubber particles were: fineness modulus 2.7, density 1190 kg/m^3^, and bulk density 365 kg/m^3^. The thickening agent used in this research was hydroxypropyl methylcellulose, with a mixture amount of 0.04% (in mass of binding material). The polycarboxylate superplasticizer with a mixture amount of 1% (in mass of binding material) and 40% solid content was used to improve the flowability of fresh SCRLC.

### 2.2. Mixture Proportions and Specimens Preparation

Six mixture proportions of SCRLC were designed as shown in Table 2. In order to explore the effect of the amount of rubber particles on the stress–strain relationship of SCRLC, the ratio between rubber particles and sand was set as a unique variable in this study. The volume substitution percentages were 10%, 20%, 30%, 40%, and 50%, respectively.

According to GB/T 50081 [35], specimens including cubes of 100 mm × 100 mm × 100 mm and prisms of 100 mm × 100 mm × 300 mm were prepared for cubic compressive strength tests, axial compressive strength tests, elastic modulus tests, and stress–strain curve tests. Fifty-four cubic specimens were used to explore the cubic compressive strength and ninety prismatic specimens were utilized to evaluate the axial compressive strength, elastic modulus, and stress–strain curves. Nine cubes and fifteen prisms were cast for each batch. Due to the excellent flowability of fresh SCRLC, SCRLC was cast into the mold without vibration. The representative value of each test was obtained from the average value of three test values for each batch and age. All specimens were cured in a controlled environment of 20 ± 5 °C and RH > 95% until testing.

### 2.3. Testing Method

**Figure 2:**
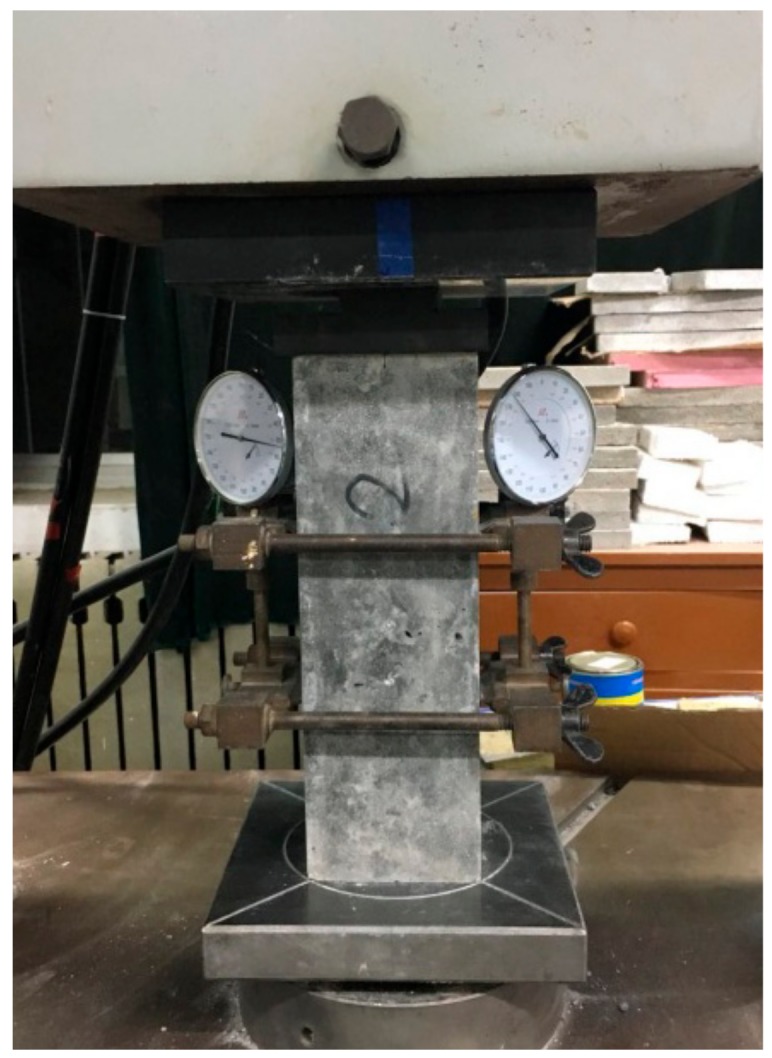
Test setup of elastic modulus.

In accordance with GB/T 50081 [35], the cubic and axial compressive strength of SCRLC were measured by a 1000 kN computer-controlled electro-hydraulic servo universal testing machine at a loading rate of 3.0 kN/s after 7, 28, and 90 days of age. The elastic modulus of SCRLC tested in this research was secant modulus at 28 days. Before testing, two dial indicators were fitted at the opposite sides of specimens for measuring the deformation (as shown in Figure 2). The load was applied by a 1000 kN computer-controlled electro-hydraulic servo universal testing machine (Changchun Kexin Test Instrument Co., Ltd., Changchun, China) at a loading rate of 3.0 kN/s. The cyclic loading with an upper limit of one-third of the ultimate load and a lower limit of 0.5 MPa was loaded six times. The first three cycles of readings were discarded and the elastic modulus was calculated from the mean of the last three cycles of readings by Equations (1) and (2):

(1) $$E_{c}=\frac{F_{a}-F_{0}}{A}\times \frac{L}{\Delta n},$$

(2) $$\Delta n=L_{a}-L_{0},$$

where *E_c_* is the elastic modulus (MPa), *F_a_* is one-third of the ultimate load (N), *F_0_* is the initial load when the stress is 0.5 MPa (N), *A* is the compression area of specimens (mm^2^), *L* is the measurement range (mm), Δ*n* is the longitudinal deformation of the specimen (mm), *L_a_* is the compression deformation corresponding to *F_a_* (mm), and *L_0_* is the compression deformation corresponding to *F_0_* (mm). 

**Figure 3:**
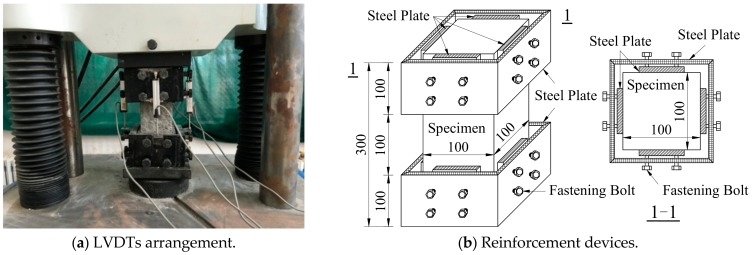
Test setup of stress–strain curves. LVDT: linear variable displacement transducer.

The stress–strain curve experiments were conducted by a 1000 kN computer-controlled electro-hydraulic servo universal testing machine with a constant rate of loading displacement 0.002 mm/s at 28 days of age. Four linear variable displacement transducers (LVDTs) (manufacturer, city, country) were placed at four sides of specimens to measure the longitudinal deformation (Figure 3). The load was recorded by the test machine and the longitudinal deformation was collected by LVDTs which were connected to a DH3820 data acquisition system for storing the data during testing in a computer. Restricted by the insufficient stiffness of the test machine, in order to prevent sudden damage and acquire a stable descending stage of the stress–strain curve, two reinforcement devices were affixed to the specimens, one on each side. This could prevent the premature destruction of both ends of the specimens caused by the cyclo-hoop effect and ensure the stress–strain curves were complete. The measurement range of longitudinal deformation was set as 100 mm. The test was terminated when the collected data could draw the whole stress–strain curve.

## 3. Results and Discussion

### 3.1. Compressive Strength of SCRLC

**Figure 4:**
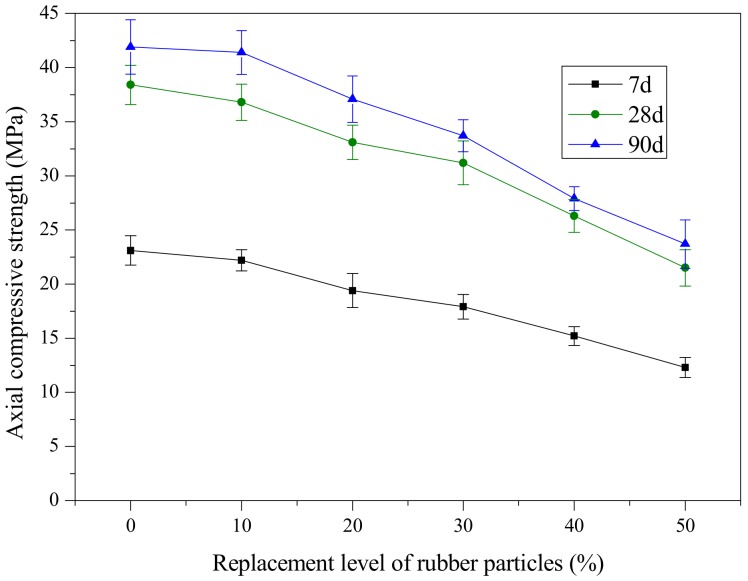
Variation in axial compressive strength with replacement level of rubber particles.

**Figure 5:**
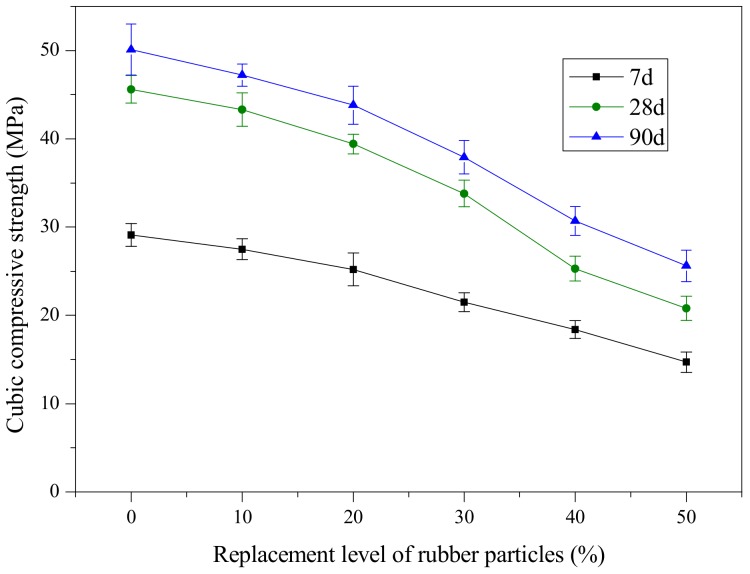
Variation in cubic compressive strength with replacement level of rubber particles.

Figure 4 and Figure 5 present the variation of the mean value of axial compressive strength and cubic compressive strength of SCRLC with various substitution percentages of rubber particles at 7, 28 and 90 days of aging. Results indicated that similar variations in axial and cubic compressive strength tendency with rubber particles substitution percentage were obtained at 7, 28, and 90 days. Both the axial and cubic compressive strength decreased as the rubber particles substitution percentage increased. Compared with river sand, rubber particles have inferior strength and poorer interface properties with hardened cement paste [9]. This might explain the above phenomenon.

**Figure 6:**
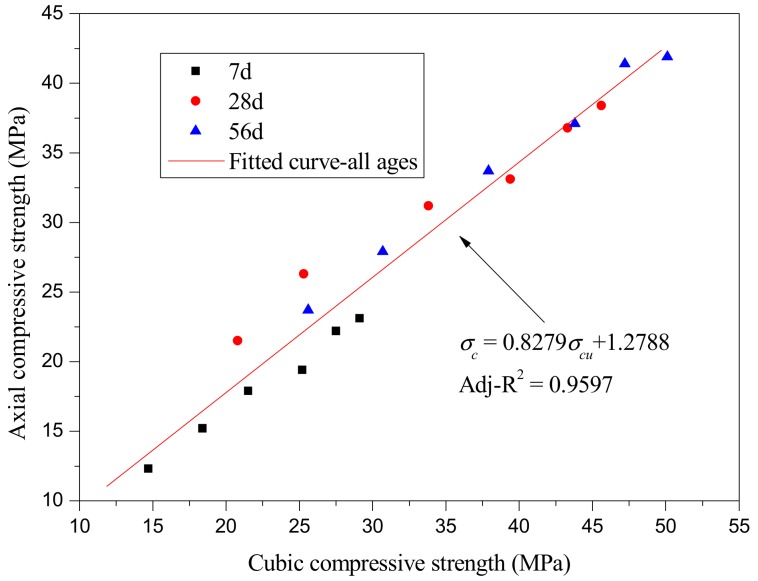
The relationship between axial and cubic compressive strength.

Based on the experimental results, the relationship between the axial and cubic compressive strength of SCRLC are given in Figure 6. It could be observed that the axial compressive strength approximately followed a linear relationship with cubic compressive strength, as expressed by Equation (3):

(3) $$\sigma_{cp}=0.8279\sigma_{cu}+1.2788,$$

where σ_cp_ is axial compressive strength (MPa) and σ_cu_ is cubic compressive strength (MPa).

Equation (3) indicates that the ratio between the axial and cubic compressive strength of SCRLC was basically in accordance with that of NC (i.e., 0.7–0.92) [36].

### 3.2. Typical Failure Pattern of Prisms 

**Figure 7:**
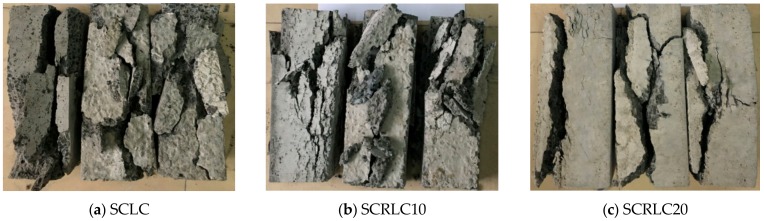

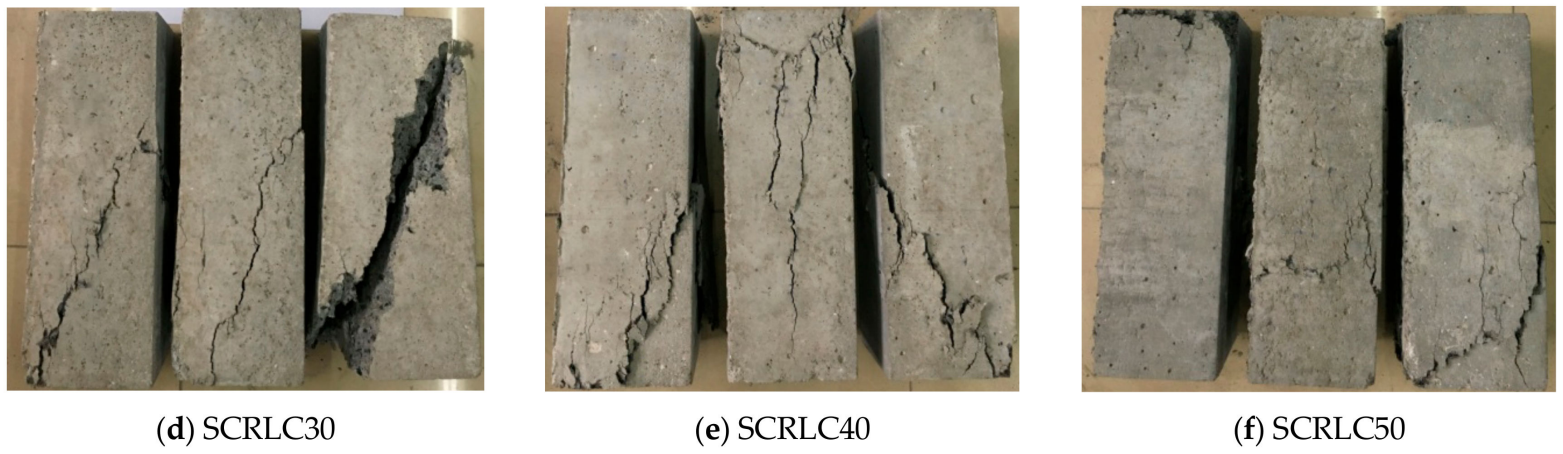
Typical failure patterns of prism specimens.

Figure 7 shows the typical failure patterns of prism specimens with different mixtures at 28 days by axial compressive strength tests. The failure patterns were quite different from each other and could be roughly divided into two categories. When the rubber particles substitution percentage was lower than 30%, the failure patterns were typical brittle failure. After attaining ultimate load during loading process, a sudden noise was heard and the load dropped rapidly. The specimens were broken into several small pieces. The failure characteristics of SCRLC changed significantly. As the rubber particles substitution percentage increased—especially as it exceeded 30%–as shown in Figure 7d–f. The specimens had a tendency to experience ductile damage. After reaching ultimate load, the loading declined but the rate of descending loading decreased. Although the cracks appeared on the surface of specimens, the specimens still had bearing capacity until the cracks penetrated the entire specimens. The variation of SCRLC failure patterns was similar to that of crumb rubber concrete (CRC) [37,38]. The higher the rubber particles substitution percentage in SCRLC, the more obvious the ductile failure characteristics. This might be mainly due to the better deformation and energy absorption abilities of rubber particles, which played a positive role in improving the toughness of SCRLC. Our results indicate that utilizing rubber particles to replace sand in SCRLC is a feasible measure to reduce the brittleness of SCRLC.

### 3.3. Uniaxial Stress–Strain Curves

**Figure 8:**
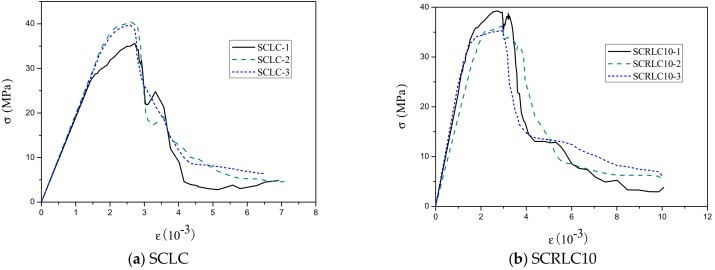

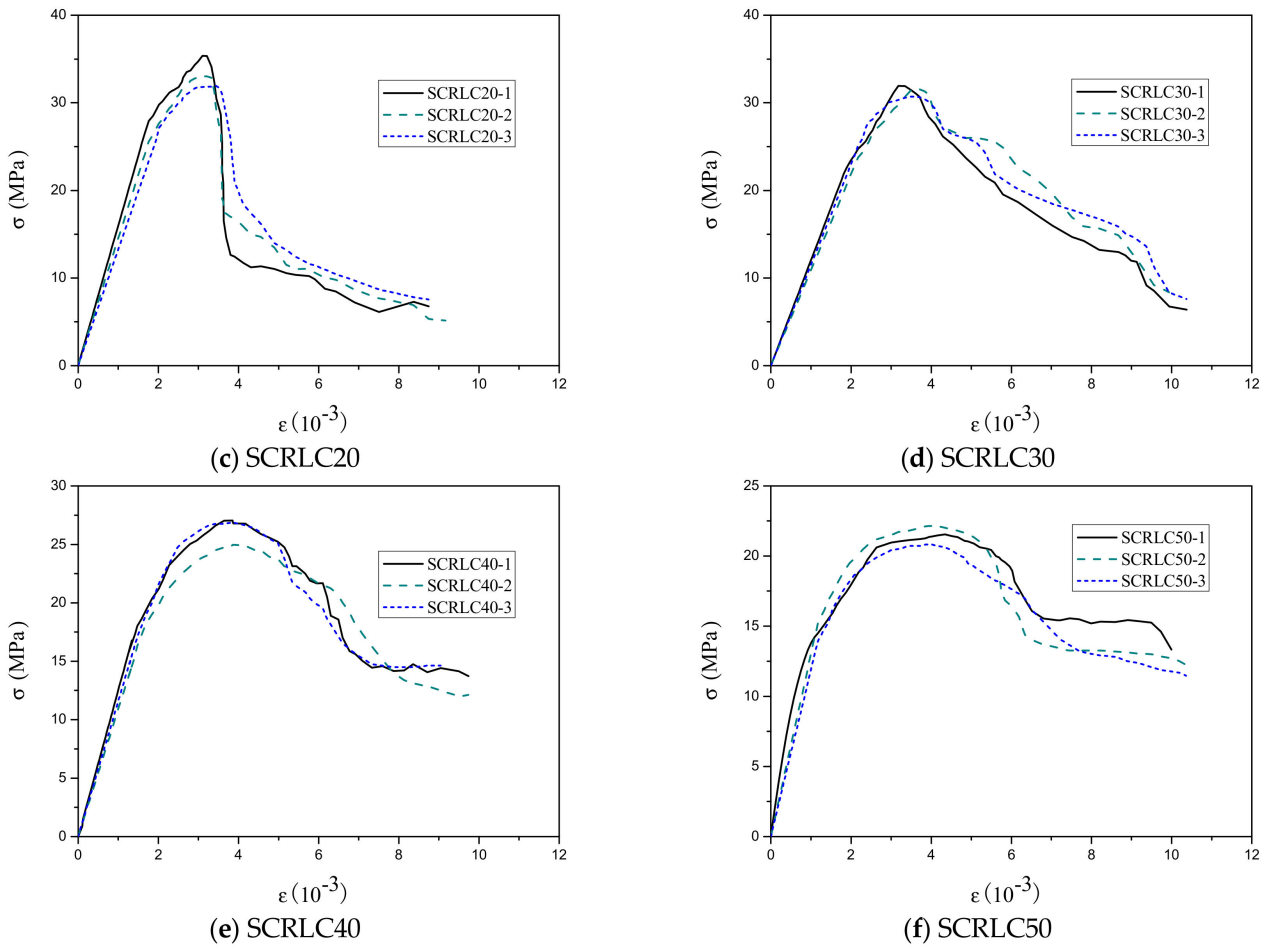
Uniaxial stress–strain curves.

**Figure 9:**
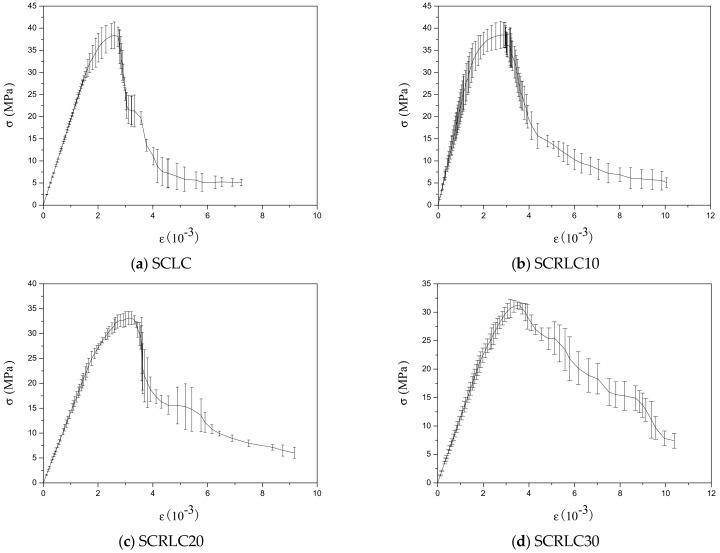

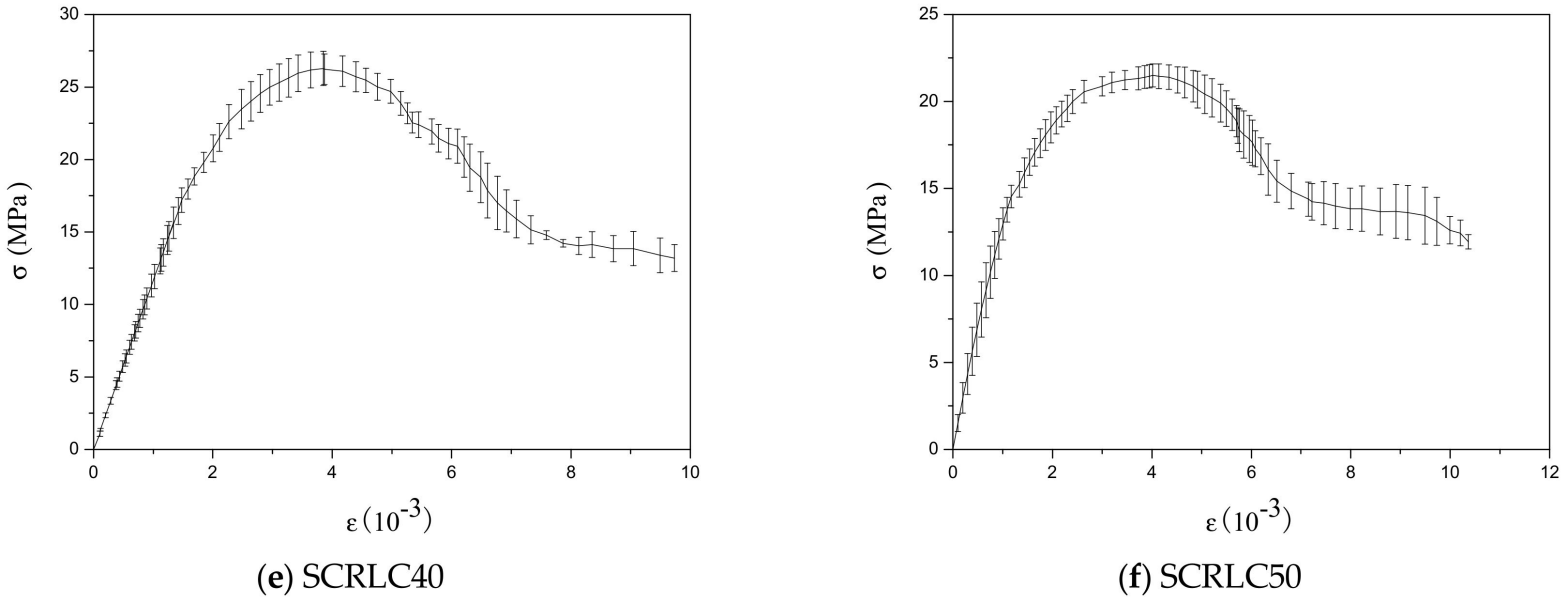
Means and standard deviations of stress–strain curves.

**Figure 10:**
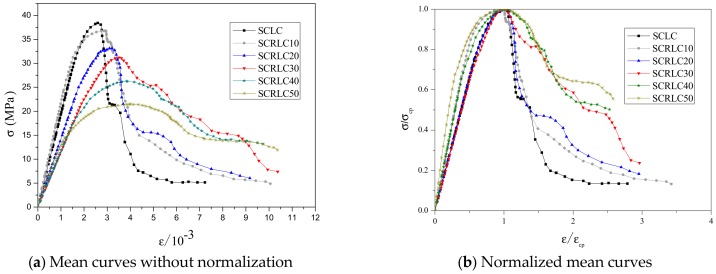
Mean stress–strain curves of SCRLC series.

The uniaxial stress–strain curves of SCRLC obtained from experiments are presented in Figure 8. Each type of mixture had three uniaxial stress–strain curves with a similar shape. The statistical results including means and standard deviation of stress–strain curves of SCRLC are shown in Figure 9. It could be seen that the standard deviations of the descending stage of the stress–strain curves were greater than those of the ascending stage. This means that the data in the descending stage were more dispersed than in the ascending stage. This might be mainly ascribed to the fact that the specimens in the descending stage of the stress–strain curves were beyond the elastic regime, and plenty of cracks occurred. Figure 10 expresses the mean stress–strain curves of the SCRLC series. From the six SCRLC stress–strain curves shown in Figure 10b, it can be seen that each type of SCRLC had similar ascending stages which increased approximately linearly, but the descending stages were different. The descending stage of the stress–strain curves of SCRLC with rubber particles substitution percentage under 30% were much steeper than in specimens with rubber particles substitution percentage over 30%. This indicates that the specimens with rubber particles substitution percentage under 30% exhibited greater brittleness than those with substitution percentage over 30%, which is consistent with the test phenomena elaborated in Section 3.2. In general, the descending stage of SCRLC stress–strain curves became softer as the rubber particles substitution percentage increased, which is in keeping with the variation seen in CRC [37,38].

The characteristic indices of the SCRLC including peak stress, peak strain, elastic modulus, and peak secant modulus acquired from the stress–strain curves are listed in Table 3. As the rubber particles substitution percentage increased, peak stress, elastic modulus, and peak secant modulus decreased, while peak strain increased. As rubber particles substitution percentage increased from 0% to 50%, the peak strain increased from 0.0025926 to 0.0040162, with peak stress deceasing from 38.4 to 21.5 MPa, and elastic modulus and peak secant modulus reducing from 26.8 to 15.5 GPa and 14.5 to 5.4 GPa, respectively. The greater the fraction of rubber particles in the SCRLC, the better the deformation and toughness properties, which is in accordance with the variation seen in CRC [38].

Compared with LC [39], despite the same compressive strength, the peak strain of SCRLC was much larger. The ratio of peak strain between SCRLC and LC increased as the peak stress decreased. This was mainly due to the fact that as the peak stress decreased, the peak strain of SCRLC increased while the peak strain of LC dropped.

**Figure 11:**
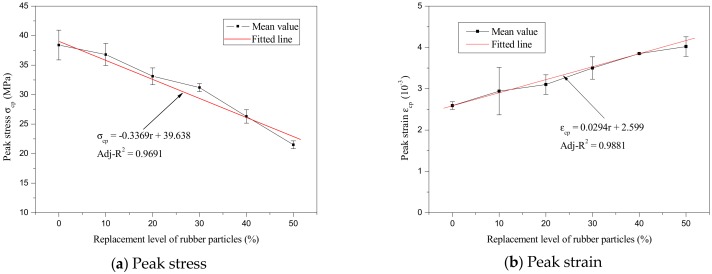
Variation in peak stress and peak strain with replacement level of rubber particles.

**Figure 12:**
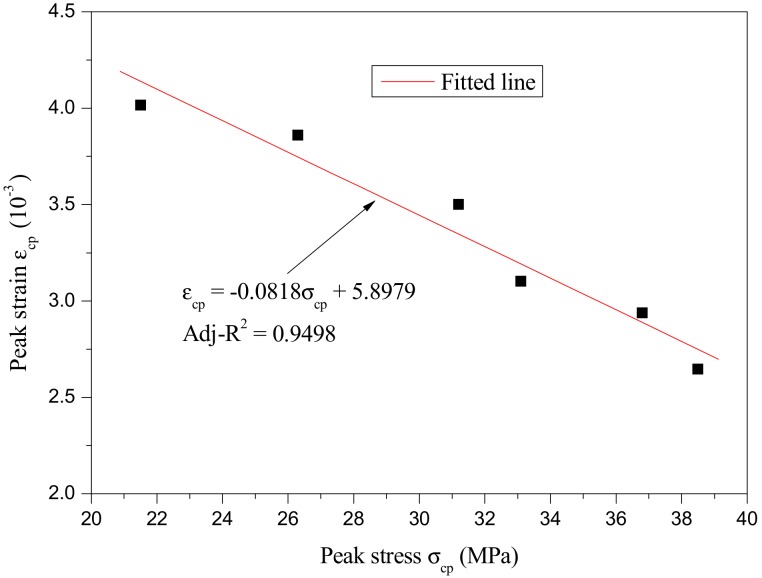
The relationship between peak strain and peak stress.

Based on the above experimental data, the variations of peak stress and peak strain with rubber particles substitution percentage are described in Figure 11. Meanwhile, the relationship between the peak strain and peak stress is described in Figure 12. As can be seen in Figure 11 and Figure 12, the relationship between peak stress and rubber particles substitution percentage, peak strain and rubber particles substitution percentage, and peak strain and peak stress were all approximately linear; the regression formulas are as follows:

(4) $$\sigma_{cp}=-0.3369r+39.638,$$

(5) $$\varepsilon_{cp}=0.0294r+2.599,$$

(6) $$\varepsilon_{cp}=-0.0818\sigma_{cp}+5.8979,$$

where σ_cp_ is peak stress (MPa), ε_cp_ is peak strain (10^−3^), and *r* is the rubber particles substitution percentage (%).

### 3.4. Stress–Strain Relationship

#### 3.4.1. Existing Models

Many models have been proposed to depict the stress–strain relationship of NC in previous studies. The most widely used models are Eurocode, Chinese code, and Guo’s model. Because the shape of experimental SCRLC stress–strain curves were similar to that of NC, these three models were utilized to depict the stress–strain relationship of SCRLC tentatively in this study. Through comparative analysis, the most applicative model was chosen to depict the stress–strain relationship of SCRLC.

(1) Chinese Code

According to GB 50010 [40], the stress–strain relationship of NC is given as follows:

(7) $$\sigma =\left(1-d_{c}\right)E_{c}\varepsilon ,$$

where *σ* is the compressive stress at each point (MPa); *E_c_* is the elastic modules (MPa); *ε* is the strain corresponding to compressive stress at each point; and *d_c_* is the damage evolution parameter under uniaxial compression, and can be calculated by the following formula:

(8) $$d_{c}=\left\{\begin{array}{l} 1-\frac{\rho_{c}n}{n-1+x^{n}}\;\;\;\;\;x\leq 1\\ 1-\frac{\rho_{c}}{\alpha_{c}{\left(x-1\right)}^{2}+x}\;\;\;\;x>1 \end{array}\right.,$$

where $\rho_{c}=\frac{\sigma_{cp}}{E_{c}\varepsilon_{cp}}$, $n=\frac{E_{c}\varepsilon_{cp}}{E_{c}\varepsilon_{cp}-\sigma_{cp}}$, $x=\frac{\varepsilon }{\varepsilon_{cp}}$, *σ_cp_* is the peak stress (MPa, and *ε_cp_* is the peak strain corresponding to the peak stress. *α_c_* is a parameter related to the descending stage of the stress–strain curve under uniaxial compression and can be determined by Equation (9) [41]:

(9) $$\alpha_{c}=0.157\sigma_{cp}^{0.785}-0.905.$$

(2) Eurocode

Based on BS EN 1992–1–1 [42], the stress–strain relationship of concrete including ascending and descending stages is described by one formula, as follows:

(10) $$\frac{\sigma }{\sigma_{cp}}=\frac{k\cdot x-x^{2}}{1+\left(k-2\right)x},$$

where $x=\varepsilon /\varepsilon_{cp}$ and $k=1.05E_{c}\cdot \left|\varepsilon_{cp}\right|/\sigma_{cp}$.

(3) Guo’s model

Guo’s model was proposed by Guo et al. [43] and considers that the stress–strain relationship of concrete should be divided into two stages to be described by their respective formulas (i.e., ascending and descending stages). Guo’s model is expressed as follows:

(11) $$y=\left\{\begin{array}{l} ax+(3-2a)x^{2}+(a-2)x^{3}\;\;\;\;0\leq x\leq 1\\ \frac{x}{b{\left(x-1\right)}^{2}+x}\;\;\;\;\;\;\;\;\;\;\;1\leq x \end{array}\right.,$$

where $x=\varepsilon /\varepsilon_{cp}$, $y=\sigma /\sigma_{cp}$, $a=E_{0}/E_{P}$, *E_0_* is the initial tangential modulus (GPa), *E_p_* is the peak secant modulus (GPa), and *b* is a parameter related to the descending stage of the stress–strain curves.

**Figure 13:**
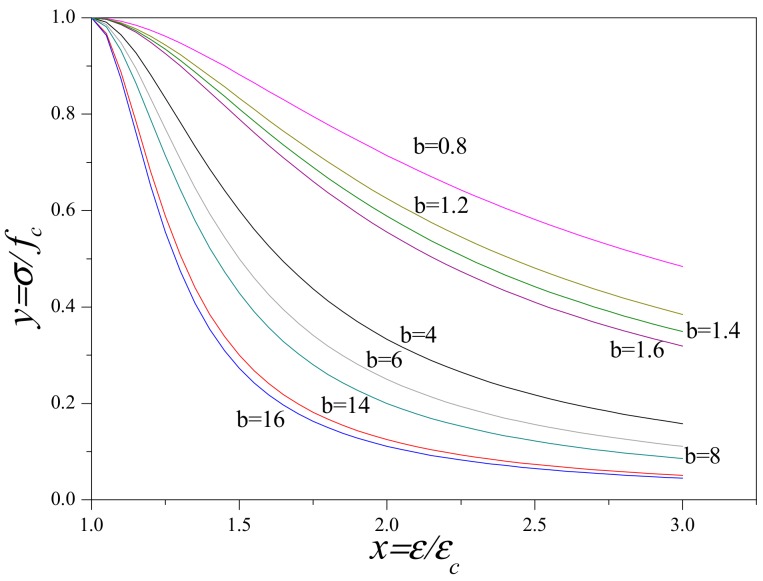
Descending stages of stress–strain curves at different *b* values.

From Equation (11), it can be deduced that if *b* was equal to 0, *y* would always be equal to 1 regardless of the value of *x*. At this moment, the descending stage of stress–strain curve becomes a straight line which is parallel to the *x*-axis. This meant that the stress remains constant as the strain increases. The material is equivalent to an ideal plastic material. Meanwhile, if *b* tends to infinity, the load drops to 0 abruptly when the strain exceeds peak strain. The material is equivalent to a complete brittle material. Based on the different *b* values, various descending stages of concrete stress–strain curves would be obtained as shown in Figure 13.

According to the test results acquired from stress–strain curves, the parameter *a* could be calculated by Equation $a=E_{0}/E_{P}$ and the parameter *b* could be confirmed by comparison of the shape of descending stage of stress–strain curves between experimental results and Figure 13. In this research, the *E_0_* was approximately equal to *E_c_*. After analysis, the values of parameters *a* and *b* were ascertained as listed in Table 4.

#### 3.4.2. Comparison Results

**Figure 14:**
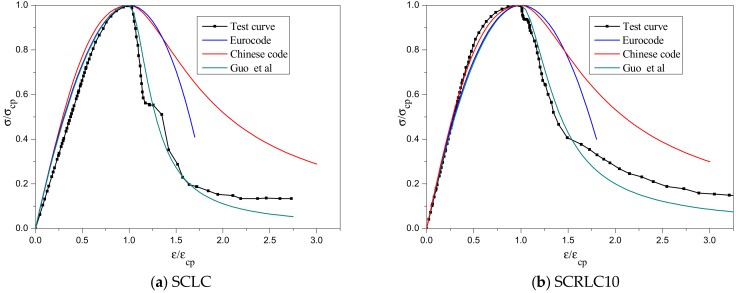

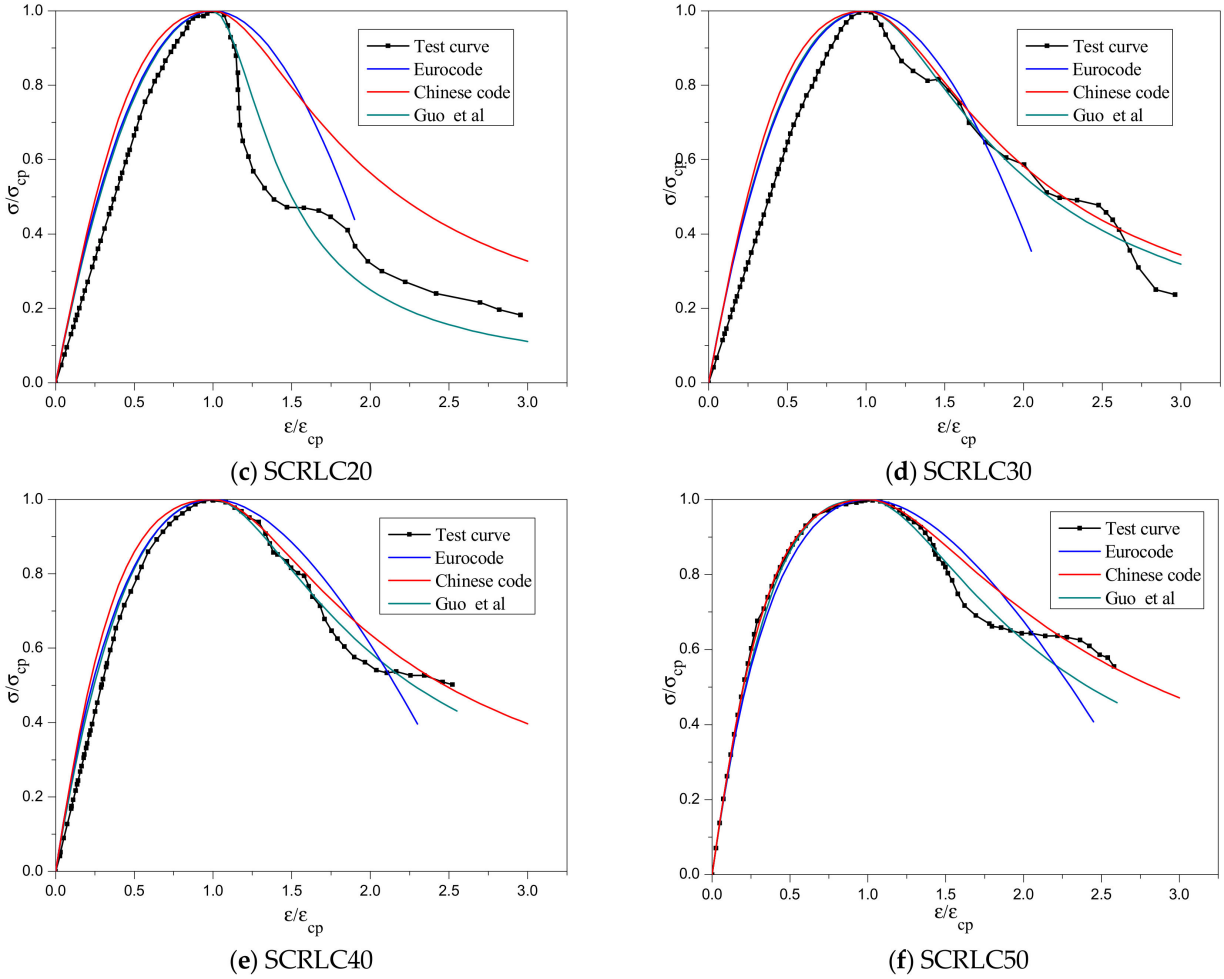
Comparison of stress–strain curves of SCRLC obtained from existing stress–strain models.

The comparison of SCRLC stress–strain curves obtained from existing stress–strain models is presented in Figure 14. As can be seen, all three models had a similar prediction of the ascending stage of stress–strain curves, which were also approximately in keeping with the experimental results. However, the descending stages of the stress–strain curves predicted by the three models were quite different from each other. When the rubber particles substitution percentage was lower than 30%, the prediction results of Eurocode and Chinese code were larger than experimental results, whereas the prediction results of Guo’s model coincided well with the experimental results. When the rubber particles substitution percentage was greater than 30%, the stress–strain curves predicted by all three models had a similar tendency and shape. In comparison, the prediction result of Guo’s model was much closer to the experimental results. Overall, Guo’s model was more suited to describe the stress–strain curve of SCRLC on account of the reasonable selection of parameter *b*.

#### 3.4.3. Parameters Analysis 

**Figure 15:**
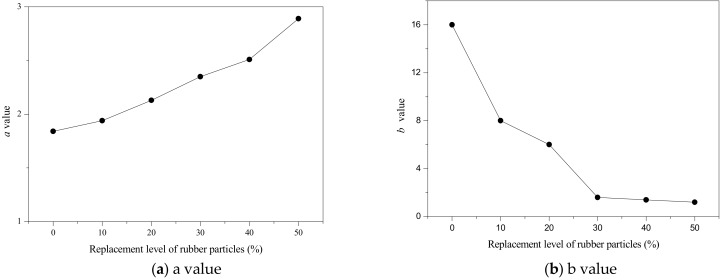
Variation of *a* and *b* values with replacement level of rubber particles.

The variation of parameters *a* and *b* with rubber particles substitution percentage is shown in Figure 15. As can be seen, with increasing rubber particles substitution percentage in SCRLC, the *a* value increased while the *b* value decreased. The variation of the *a* value indicated that the reduction of the peak secant modulus was greater than the initial tangential modulus as the rubber particles substitution percentage increased. This means that the proportion of plastic deformation in the total deformation increased and the plastic deformation capacity near peak loading was enhanced. The *b* value was a constant which determined the shape of descending stage of stress–strain curve. The greater the *b* value was, the steeper the descending stage of the stress–strain curve would be. With increasing rubber particles substitution percentage, the *b* value dropped dramatically at first and then declined slightly. The changing point occurred at a rubber particles substitution percentage of 30%, meaning that the brittleness of SCRLC decreased remarkably when the rubber particles substitution percentage was larger than 30%, which is in keeping with the experimental results.

**Figure 16:**
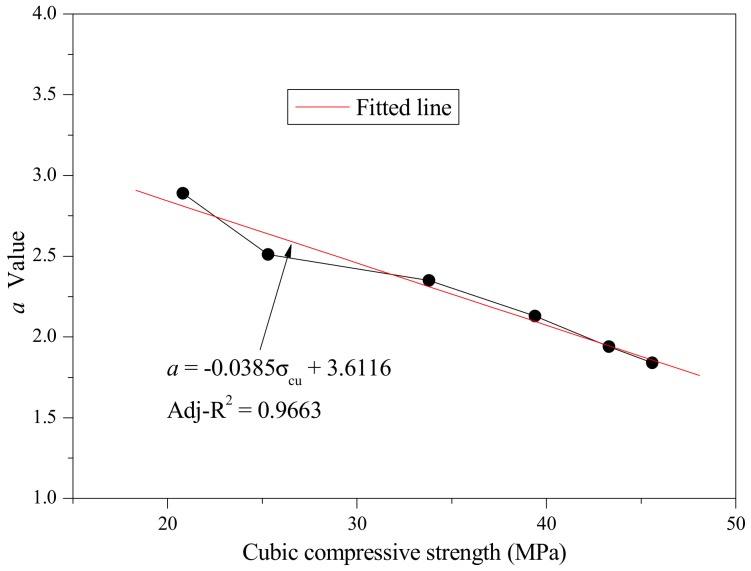
Fitted curve of the *a* value and cubic compressive strength.

**Figure 17:**
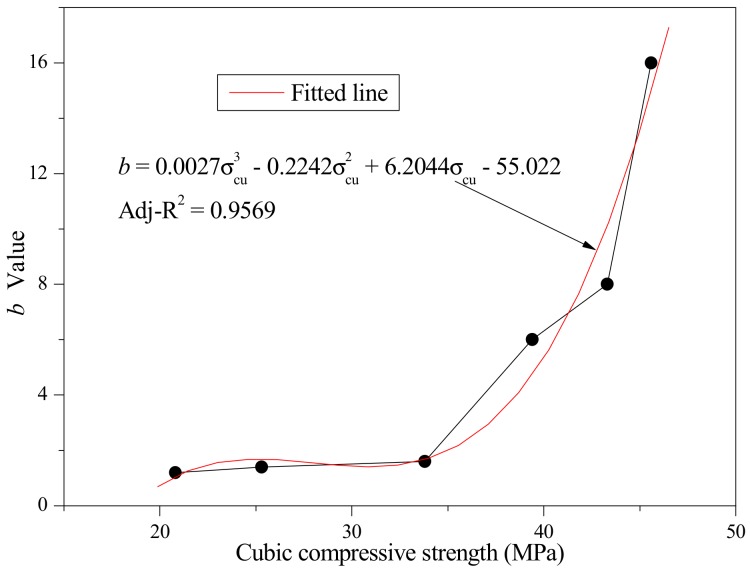
Fitted curve of the *b* value and cubic compressive strength.

By statistical analysis, the relationships between the *a* value and the cubic compressive strength and *b* value and cubic compressive strength were established as shown in Figure 16 and Figure 17, respectively. The relationship between the *a* value and cubic compressive strength was almost linear, while the relationship between the *b* value and cubic compressive strength was a curve described by a cubic equation with one unknown. The regression equations are expressed as follows:

(12) $$a=-0.0385\sigma_{cu}+3.6116,$$

(13) $$b=0.0027\sigma_{cu}^{3}-0.2242\sigma_{cu}^{2}+6.2044\sigma_{cu}-55.022.$$

#### 3.4.4. Stress–Strain Relationship Prediction 

**Figure 18:**
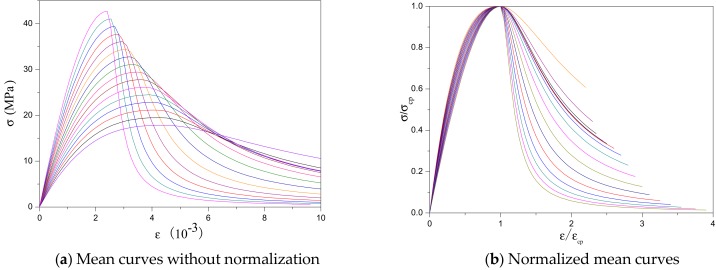
Mean stress–strain curves obtained by Guo’s model.

Combined with the relationship between cubic compressive strength and peak stress described by Equation (3), peak stress and peak strain described by Equation (6), Guo’s model described by Equation (11), the parameter a and cubic compressive strength by Equation (12), and the parameter b and cubic compressive strength by Equation (13), the mean stress–strain curves of SCRLC with cubic compressive strength varying from 20 to 50 MPa were calculated and are presented in Figure 18. As can be seen from Figure 18a, a decrease of cubic compressive strength would result in an increase of peak strain, elastic modulus, and residual strength, while it would also lead to a softer descent of the SCRLC stress–strain curve. With increasing cubic compressive strength from 20 to 50 MPa, the peak strain dropped from 0.00443885 to 0.00240718 with a reduction of about 46%. Guo’s model would achieve a good prediction of the SCRLC stress–strain relationship with a reasonable parameter selection.

## 4. Conclusions

In this paper, uniaxial compressive experiments and analyses were conducted to evaluate the stress–strain relationship of SCRLC. Based on the experimental results, the failure pattern of specimens and the characteristics of stress–strain curves were analyzed and a prediction model of the SCRLC stress–strain relationship was proposed. The main findings were drawn as follows:

(1) The failure patterns of SCRLC prism specimens were significantly affected by the content of rubber particles in the SCRLC. With increasing rubber particles substitution percentage, the failure patterns gradually transformed from brittle to ductile failure.
(2) As the rubber particles substitution percentage increased from 0% to 50%, the peak strain increased from 0.0025926 to 0.0040162 while peak stress, elastic modulus, and peak secant modulus deceased from 38.4 to 21.5 MPa, 26.8 to 15.5 GPa, and 14.5 to 5.4 GPa, respectively.
(3) Six types of SCRLC had similar variation trends in the ascending stage of stress–strain curves, which were all approximately linear. However, the descending stages of the stress–strain curves were different, especially when the rubber particles substitution percentage was greater than 30%. The descending stages of stress–strain curves of SCRLC with rubber particles substitution percentage under 30% were much steeper than those with substitution percentages over 30%. The results suggest that the deformation and toughness properties of SCRLC would be improved significantly with the incorporation of rubber particles.
(4) In a comparison of three widely-used models for describing the stress–strain relationship of SCRLC, Guo’s model was much more suitable for describing the full stress–strain curves, including ascending and descending stages. Based on the established relationships between characteristic parameters, the different strength levels of the stress–strain relationship of SCRLC would be predicted accurately by Guo’s model.

## Figures and Tables

**Table 1:** Chemical compositions of ordinary Portland cement and fly ash.

Mineral Compositions	Content/%								
	CaO	SiO_2_	Al_2_O_3_	Fe_2_O_3_	MgO	SO_3_	K_2_O	Na_2_O	Loss on Ignition
**Ordinary Portland Cement**	62.45	20.18	4.91	3.88	2.67	2.14	0.47	0.29	2.05
**Fly Ash**	5.31	48.92	26.27	5.86	0.84	1.21	0.79	0.22	3.60

**Table 2:** Mix proportions for self-compacting rubber lightweight aggregate concrete (SCRLC).

Type of Concrete	Replacement (by Volume)	Weight per Cubic Meter (kg/m^3^)							
		Cement	Fly Ash	Rubber Particles	Sand	Shale Ceramsite	Thickener	Water Reducer	Water
SCLC	0%	425	85	0	700	610	0.204	5.1	179
SCRLC10	10%	425	85	31	630	610	0.204	5.1	179
SCRLC20	20%	425	85	62	560	610	0.204	5.1	179
SCRLC30	30%	425	85	93	490	610	0.204	5.1	179
SCRLC40	40%	425	85	124	420	610	0.204	5.1	179
SCRLC50	50%	425	85	155	350	610	0.204	5.1	179

**Table 3:** Characteristic indices of SCRLC under uniaxial loading.

NO.	Peak Stress σ_cp_ (MPa)	Peak Strain ε_cp_ (10^−3^)	Elastic Modulus E_c_ (GPa)	Peak Secant Modulus E_cp_ (GPa)
SCLC	38.4	2.5926	26.8	14.5
SCRLC10	36.8	2.9378	24.3	12.5
SCRLC20	33.1	3.1017	22.7	10.7
SCRLC30	31.2	3.5011	19.9	8.9
SCRLC40	26.3	3.8604	18.1	6.8
SCRLC50	21.5	4.0162	15.5	5.4

**Table 4:** The values of parameters *a* and *b*.

Parameters	SCLC	SCRLC10	SCRLC20	SCRLC 30	SCRLC 40	SCRLC 50
*a*	1.84	1.94	2.13	2.35	2.51	2.89
*b*	16	8	6	1.6	1.4	1.2
